# The iron-energy metabolism axis in Alzheimer’s pathogenesis: from mechanisms to interventions

**DOI:** 10.1038/s41420-026-03034-w

**Published:** 2026-04-22

**Authors:** Zhenyou Zou, Jia Chen, Jing Li, Yongfeng Chen

**Affiliations:** 1 Guangxi Zhuang Autonomous Region Brain Hospital, Liuzhou, China; 2 School of Medicine, Taizhou University, Taizhou, China; 3 Osaka Metropolitan University Graduate School of Medicine, Osaka, Japan; 4 School of Medicine, Zhejiang University, Hangzhou, China; 5 Institute of Basic Medicine, North Sichuan Medical College, Nanchong, China

**Keywords:** Cellular neuroscience, Mechanisms of disease

## Abstract

Alzheimer’s disease (AD) is a neurodegenerative disorder with a complex, multifactorial pathogenesis. Growing evidence implicates disturbances in cellular energy metabolism and iron dyshomeostasis as interlinked contributors to pathology. Within this framework, iron accumulation may act as an upstream regulator in certain contexts and stages, while in others it emerges downstream and amplifies ongoing injury. As iron is an essential cofactor for mitochondrial respiration and the tricarboxylic acid cycle, iron imbalance can compromise ATP production and disrupt glucose metabolism, exacerbating neuronal energy deficits. The interplay among iron accumulation, oxidative stress, and neuroinflammation can create vicious cycles that reprogram cellular metabolism and disrupt the critical metabolic coupling between neurons and glial cells. This review synthesizes recent advances in understanding the iron–energy metabolism axis in AD, delineates mechanisms by which iron imbalance precipitates mitochondrial dysfunction and glucose metabolic impairments, and evaluates how these deficits synergize with neuroinflammation and proteinopathy across disease stages. Finally, we appraise emerging therapeutic strategies targeting iron overload and metabolic pathways, discuss their stage-dependent risks and benefits, and outline the need for biomarker-guided approaches to optimize patient selection and treatment timing.

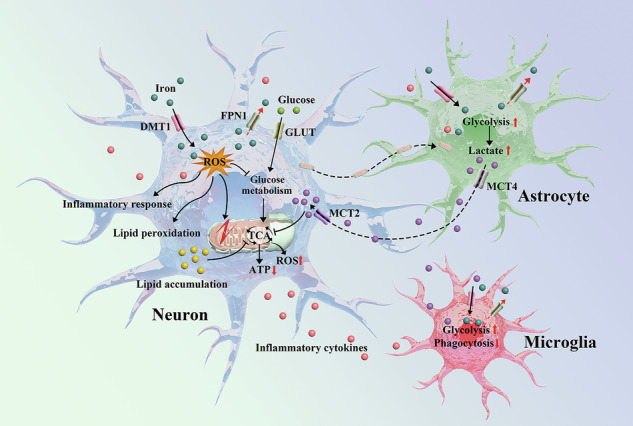

## Facts

- AD is driven by interconnected iron dyshomeostasis and bioenergetic failure, wherein iron overload acts as an upstream trigger of mitochondrial damage, metabolic dysfunction, and neuroinflammation.
- Excessive iron promotes oxidative stress via Fenton chemistry, impairing electron transport chain function, glucose metabolism, and insulin signaling, thereby exacerbating neuronal energy deficit.
- Iron overload and metabolic dysfunction engage in a vicious cycle with neuroinflammation, thereby exacerbating energy deficits and driving AD progression.
- While iron chelators and metabolic interventions show preclinical promise, their efficacy is limited by poor blood–brain barrier penetration, side effects, and stage-dependent variability.

## Open questions

- Is iron overload a primary trigger or a secondary consequence in AD progression, and does this role shift across different disease stages?
- What is the precise mechanistic link between iron dyshomeostasis, Aβ/tau pathology, and insulin resistance in the AD brain?
- Does iron overload directly reprogram glial cell metabolism, and how does this metabolic reprogramming disrupt neuron-glia metabolic coupling to exacerbate energy deficits?
- How can we optimize the timing, brain delivery, and patient stratification for iron-chelation therapy to maximize efficacy and minimize side effects?

## Introduction

Alzheimer’s disease (AD) is a prevalent neurodegenerative disorder marked by progressive memory loss, cognitive decline, and behavioral changes. Although its precise etiology remains elusive, growing evidence implicates disturbances in iron homeostasis as an integral component of a broader pathogenic network. Iron, an essential trace element, supports critical central nervous system functions, including oxygen transport, electron transfer, cellular respiration, DNA/RNA/protein synthesis, neurotransmitter production, myelination, and mitochondrial function [1].

Imaging and histopathological studies have consistently report iron accumulation in cognition-relevant regions such as the hippocampus and cortex. Elevated cortical iron levels are associated with accelerated cognitive decline in individuals with AD pathology [2, 3]. Although the precise role and causal direction of iron accumulation in AD require further elucidation, converging evidence indicates that excess labile iron can catalyze Fenton reactions, generating reactive oxygen species (ROS) and contributing to oxidative stress and neuroinflammation—hallmark features of AD [4]. Iron also participates in ferroptosis, an iron-dependent regulated cell death pathway driven by lipid peroxidation and compromised antioxidant defenses [3]. Furthermore, iron interacts with amyloid-β (Aβ) and tau, potentially modulating their aggregation and toxicity; conversely, proteinopathy, neuroinflammation, and vascular dysfunction can disrupt iron regulation and promote its retention [3, 5].

Notably, emerging studies underscore that iron overload and its associated oxidative stress are central not only to neuroinflammation but also to the disruption of brain energy metabolism—through mitochondrial dysfunction and downregulation of key metabolic enzymes [3, 4, 6]. Critically, energy metabolic imbalance and neuroinflammation are not isolated events; rather, they may engage in a self-reinforcing vicious cycle that propels neurodegenerative progression [6]. On one hand, disturbances in cerebral energy metabolism can directly trigger or exacerbate neuroinflammatory responses [7]. On the other hand, sustained neuroinflammation can, in turn, disrupt normal cellular energetics and impair the metabolic coupling between neurons and glia [8]. This bidirectional, feed-forward pathological loop is increasingly recognized as a key mechanism underlying the progression of neurodegenerative disorders, including AD.

In this review, we synthesize evidence on the iron–energy metabolism axis in AD and examine how iron dyshomeostasis may drive mitochondrial dysfunction, metabolic stress, and inflammatory amplification. These interconnected processes likely heighten synaptic vulnerability and accelerate disease progression, while also informing the timing and targets of potential therapeutic strategies.

## Regulation of iron homeostasis in the brain

Iron is the most abundant metal element in the brain, and iron homeostasis is essential for maintaining normal brain function. Brain iron metabolism is strictly regulated, primarily through the coordinated action of various iron metabolism-related proteins [9]. Disruption of brain iron metabolism leads to iron accumulation, which promotes the formation of free radicals and oxidative stress, damaging neuronal function and potentially resulting in neuronal death [10]. Increasing evidence suggests that abnormal iron metabolism is closely associated with the onset and progression of neurodegenerative diseases.

### Molecular network for the regulation of iron homeostasis

Brain iron uptake is regulated by transferrin receptor 1 (TfR1) and divalent metal transporter 1 (DMT1). Extracellular Fe²⁺ is transported into cells through DMT1, while Fe³⁺ binds to transferrin (Tf) and enters cells via endocytosis after binding to TfR1, forming endosomes. Proton pumps in the endosomes dissociate Fe³⁺ from the Tf/TfR1 complex, reducing it to Fe²⁺, which is then transported into the cytoplasm via DMT1 [11]. In the central nervous system, some Fe²⁺ is metabolized in the neuronal cytoplasm, while the rest is stored as Fe³⁺ in ferritin. When iron levels are low, ferritin is degraded by lysosomes to release iron, fulfilling neuronal needs [12].

Iron metabolism in the brain is subject to multiple regulatory mechanisms, among which hepcidin acts as a significant negative regulator by controlling the expression of ferroportin (FPN) to regulate iron transport [11]. Iron homeostasis in neurons is primarily regulated at the transcriptional level of mRNAs involved in iron metabolism. The mRNAs encoding TfR1, ferritin, FPN1, and DMT1 all contain a special amino acid sequence known as an iron-responsive element (IRE). Iron regulates the expression of these iron-related proteins by influencing the binding of IRE to iron regulatory proteins (IRPs), thereby maintaining intracellular iron homeostasis [13]. In the context of AD, the expression of several key molecules responsible for iron transport, storage, and homeostasis regulation is significantly altered [14–22] (Table 1), suggesting that abnormal iron metabolism may be involved in the progression of AD.

**Table 1:** Key molecules in brain iron homeostasis in AD.

Molecule	Function	Implication in AD Pathology	Ref.
Tf	Transports iron ions, mainly carries ferric iron in the blood to various tissues	Tf isoform (Man-Tf) levels are significantly increased in AD and MCI patients and highly correlate with phosphorylated-tau (p-tau)	[14]
TfR1	The receptor for Tf, mediates cellular uptake of iron	TfR1 expression increases during AD-iPS differentiation, along with total iron, labile iron, and ROS levels	[15]
FPN1	The only known iron exporter, transports iron out of cells	Increased FPN1 expression in AD-iPS reduces intracellular iron and ROS levels	[15]
DMT1	Transports divalent metal ions, including ferrous iron	Higher expression of DMT1 is linked to iron dyshomeostasis and increased Aβ generation in AD brains	[16]
Ferritin	Stores iron ions, regulates intracellular iron levels	Ferritin levels are significantly increased in AD patients, and the resulting iron dyshomeostasis is associated with disease progression	[17]
Hepcidin	Regulates iron absorption and distribution, inhibits FPN1	Serum hepcidin levels are significantly higher in AD patients, correlating with increased iron dyshomeostasis and cognitive impairment	[17]
IRPs	Regulate the expression of genes involved in iron metabolism	Iron accumulation disrupts IRP-IRE binding, upregulating APP, α-Syn, and Aβ levels, and promoting these proteins’ aggregation	[13]
Mfrn1	Responsible for iron transport into mitochondria	Knockdown of Mfrn1 in C. elegans AD models extended lifespan and decreased mitochondrial iron and ROS levels	[18]
Mfrn2	Responsible for iron transport into mitochondria	In initial AD patients, the expression of Mfrn2 is upregulated, enhancing the transport of iron into mitochondria	[19]
ABCB7	Transports Fe-S clusters from mitochondria to the cytoplasm	Downregulation of ABCB7 expression affects the release of mitochondrial Fe-S clusters, impairing mitochondrial function	[19]
ABCB8	Transports iron from mitochondria to the cytoplasm	Downregulation of ABCB8 expression affects the release of mitochondrial iron, leading to the accumulation of iron	[19]
Frataxin	Mitochondrial protein involved in iron-sulfur cluster biogenesis	In initial AD patients, frataxin transcripts are upregulated, possibly compensating for low mitochondrial iron availability	[19]
VDAC-1	Facilitates the transport of metabolites and ions across the mitochondrial membrane	VDAC1 expression is increased in the brain of AD mice and Aβ1-42-treated PC12 cells. Inhibiting VDAC1 alleviates mitochondrial dysfunction and ferroptosis in AD neurons	[20]
FtMt	Mitochondrial iron storage protein with an antioxidant role	Increased FtMt expression in the brains of AD patients may exert a protective role by reducing iron-mediated amyloid precursor protein expression and decreasing β- and γ-secretase secretion	[21]
mitoNEET	Mitochondrial protein that regulates iron and ROS homeostasis.	In mice, mitoNEET loss reduces ATP production, increases ROS, and causes inflammation, leading to neuronal damage	[22]

*ABCB* ATP-binding cassette subfamily B, *AD-iPS* AD patient-derived induced pluripotent stem cells.

### Dynamic regulation of mitochondrial iron pool

In mammalian cells, mitochondria are crucial organelles for regulating iron homeostasis. Iron can be stored in mitochondria and used for the synthesis of iron-sulfur (Fe-S) clusters and heme. Moreover, iron serves as a cofactor for various mitochondrial enzymes, participating in essential biological processes such as energy metabolism and redox reactions [23].

Various proteins are known to be involved in the regulation of mitochondrial iron metabolism, including iron uptake proteins like mitoferrin (Mfrn) and voltage-dependent anion channel-1 (VDAC-1), iron export proteins like ATP-binding cassette subfamily B member 7/8 (ABCB7/8), and iron storage proteins like mitochondrial ferritin (FtMt) [18–21]. Additionally, frataxin acts as an iron chaperone, assisting in the assembly of Fe-S clusters and efficient iron utilization, and plays a key role in protecting mitochondria from oxidative stress damage [24]. The mitochondrial membrane protein mitochondrial outer membrane protein containing the Asn-Glu-Glu-Thr (NEET) sequence (mitoNEET) is a crucial regulator of Fe-S cluster metabolism, participating in the maintenance of mitochondrial function, redox balance, and the distribution of Fe-S clusters between different cellular compartments [25]. These proteins work in coordination to maintain mitochondrial iron homeostasis.

Mitochondria, central to cellular energy metabolism, rely on a dynamic balance of iron ions for proper function. Disruption of mitochondrial iron metabolism impairs heme and Fe-S cluster synthesis, affecting cellular respiration and redox balance [26]. LeVine et al. recently demonstrated that in early-stage AD patients, mitochondrial iron import protein Mfrn2 is upregulated, while the Fe-S cluster export proteins ABCB7 and ABCB8 are downregulated, leading to abnormal iron accumulation in mitochondria [19]. Despite this, mitochondrial iron bioavailability decreases, potentially due to reduced PITRM1 activity. PITRM1 deficiency causes abnormal accumulation of Aβ and tau aggregates, which chelate iron ions, inducing functional iron deficiency. This impairs iron-dependent enzymes like cytochrome c oxidase, further disrupting mitochondrial function [19]. Furthermore, while the bioavailability of iron is reduced, its oxidative toxicity persists and may be exacerbated due to mitochondrial damage [19].

## Iron overload and mitochondrial energy metabolism crisis

Mitochondria, as the hub of cellular energy metabolism, are also key contributors to ROS generation in response to iron [26]. Excessive iron-induced ROS production damages the electron transport chain (ETC), inhibiting key enzymes in the TCA cycle and reducing ATP production. Simultaneously, ROS accumulation disrupts the mitochondrial membrane, exacerbating energy metabolism disorders by impairing mitochondrial dynamics (fusion/fission imbalance) and quality control systems, such as autophagy inhibition [26].

### Iron overload mediated mitochondrial ETC damage

The mitochondrial ETC is the core of OXPHOS and consists of Complex I (NADH dehydrogenase), Complex II (succinate dehydrogenase), Complex III (cytochrome bc₁ complex), Complex IV (cytochrome c oxidase), and Complex V (ATP synthase). These complexes generate a transmembrane proton gradient through a cascade of electron transport reactions, driving ATP synthase to synthesize ATP [27]. Complexes I, III, and IV require Fe-S clusters and/or heme as cofactors to support OXPHOS by mitochondrial ATP synthase [28].

Mitochondrial Fe-S clusters, composed of iron and sulfur atoms, are essential cofactors in ETC complexes and key enzymes of the TCA cycle, regulating electron transfer and redox reactions [28, 29]. These clusters are sensitive to iron concentration and redox state. In iron overload conditions, ROS production increases in primary cortical neurons, significantly altering several mitochondrial proteins, including Complex I. Proteomic analysis reveals that iron overload damages Fe-S clusters, inhibiting key subunits Ndufs1 and NDUFA10 in Complex I. This impairs electron transfer from NADH to ubiquinone, reducing proton translocation and ATP synthesis efficiency [30]. ROS also inactivates Fe-S cluster-dependent enzymes in the TCA cycle, such as aconitase, hindering the cycle’s function [31]. Furthermore, damage to Fe-S clusters affects mitochondrial DNA repair enzymes like BRIP1/FANCJ, exacerbating oxidative DNA damage and creating a vicious cycle [32, 33].

Heme is a vital cofactor for ETC Complexes III and IV, and its synthesis depends on ferrochelatase inserting Fe²⁺ into protoporphyrin IX [34]. In AD, disrupted heme metabolism is a key pathological feature, marked by symptoms like uncontrolled iron accumulation, decreased heme A, increased heme B, and Complex IV dysfunction [35]. Studies confirm that Aβ not only binds iron but also heme, potentially causing heme insufficiency. The combined deficiency of heme and iron disrupts ETC activity, worsening mitochondrial dysfunction. Additionally, Aβ-heme complexes exhibit peroxidase activity, making them a significant pathological feature of AD [19, 36].

### Iron overload mediated mitochondrial membrane potential collapse and energy generation impairment

The mitochondrial membrane potential (MMP), a proton motive force across the inner mitochondrial membrane, is essential for driving ETC and ATP synthesis [37]. The lipid composition of the inner mitochondrial membrane critically determines the activity/assembly of ETC complexes and cristae structure. Notably, cardiolipin, rich in polyunsaturated acyl chains, is highly susceptible to attack by iron overload-driven ROS and lipid peroxidation [37, 38].

Oxidized cardiolipin alters the fluidity and curvature of the inner mitochondrial membrane, disrupting cristae architecture and destabilizing ETC supercomplexes. This process weakens the MMP and diminishes ATP production. Furthermore, oxidized cardiolipin acts synergistically with Ca²⁺ to promote the opening of the mitochondrial permeability transition pore (MPTP), leading to proton leakage and MMP collapse [38–40]. Transient, low-conductance MPTP openings are generally associated with adaptive stress responses, whereas sustained, high-conductance openings trigger irreversible damage. The disruption of mitochondrial calcium homeostasis is a pivotal factor driving the transition from adaptive stress to irreversible injury [39, 40].

In neurons and related models, factors such as iron overload, elevated ROS, or Aβ/tau pathology can disrupt calcium homeostasis. Evidence often suggests a pattern of increased MCU activity and/or impaired NCLX function, leading to Ca²⁺ overload within the mitochondrial matrix [41–44]. Ca²⁺ activates calcium-dependent enzymes, such as calpain and calcineurin, exacerbating mitochondrial structural and functional damage [45]. Moreover, high levels of Ca²⁺ and ROS form a vicious cycle, strongly driving the MPTP towards a sustained open state. Excessive opening of the MPTP causes swelling of the mitochondrial matrix and mitochondrial outer membrane permeabilization, resulting in the release of cytochrome c from the mitochondria to the cytoplasm, which subsequently induces cell death [38–40, 46].

At this stage, if the cell maintains a basal ATP level, the released cytochrome c binds to apoptotic protease activating factor-1 in an ATP/dATP-dependent manner to form the “apoptosome,” thereby activating the caspase cascade and executing orderly, non-inflammatory programmed apoptosis. In scenarios of rapid ATP depletion, this energy-dependent apoptotic program fails to initiate [40]. Concurrently, the failure of energy-dependent Na⁺/K⁺-ATPase and calcium pumps leads to sodium and calcium influx, causing cellular swelling and exacerbating calcium overload, while also activating proteases and phospholipases, ultimately inducing necrotic death characterized by membrane rupture [40, 45, 47]. Additionally, high levels of ROS driven by iron overload can trigger ferroptosis, a caspase-independent form of programmed cell death. Its core mechanism involves iron-driven accumulation of polyunsaturated lipid peroxides and impaired function of glutathione peroxidase 4 (GPX4). Ca²⁺ overload and ROS not only promote lipid peroxidation and mitochondrial dysfunction but also weaken the cellular antioxidant defenses, thereby increasing susceptibility to ferroptosis [26, 40, 48]. Neurons, due to their high iron content, high metabolic rate, and limited capacity to replenish antioxidants, are particularly vulnerable to ferroptosis [49].

Intervention studies targeting the above mechanistic nodes confirm their therapeutic potential. On one hand, directly stabilizing mitochondrial structure and function is protective. For instance, the MPTP inhibitor Cyclosporine A can restore mitochondrial membrane potential and ATP levels in neural cells in experimental models of brain injury [50]; similarly, drugs stabilizing cardiolipin, such as Elamipretide, have been shown to preserve neuronal mitochondrial function and improve cognitive deficits in a lipopolysaccharide (LPS)-treated mouse model [51]. On the other hand, correcting the imbalance in mitochondrial calcium homeostasis is also effective. Application of the MCU inhibitor Ru360 can prevent mitochondrial calcium overload and block ROS production, thereby ameliorating iron overload-induced mitochondrial dysfunction in mouse brains [52]; in vitro experiments demonstrate that downregulating MCU alleviates ferroptosis in the HT22 murine hippocampal cell line [53]. Similarly, in neuron-related studies, pharmacologically activating NCLX (using the phosphodiesterase 2 inhibitor Bay 60-7550) to enhance calcium efflux can protect neurons from excitotoxic injury and improve learning and memory in mice [54]. These findings suggest that preventing the irreversible opening of the MPTP by maintaining mitochondrial membrane integrity and calcium homeostasis represents a crucial strategy for counteracting iron overload-associated mitochondrial damage and cell death.

### Iron overload mediates imbalance in mitochondrial dynamics and quality control

Mitochondria, dynamic organelles, regulate their morphology, quantity, size, and spatial distribution through continuous fusion and fission, ensuring mitochondrial homeostasis and activity, which is crucial for cellular energy metabolism [55]. However, in conditions like AD and iron overload, mitochondrial dynamics become significantly imbalanced [56].

According to Kang et al., during the differentiation of induced pluripotent stem cells derived from AD patients, there is an abnormal accumulation of total iron, labile iron, and ROS levels in cells. Mechanistically, increased TFR1 expression drives the upregulation of key mitochondrial fission proteins, dynamin-related protein 1 (DRP1) and fission 1 (FIS1), while suppressing the fusion proteins mitofusin 2 and optic atrophy 1 (OPA1). This imbalance in mitochondrial dynamics ultimately leads to mitochondrial impairment [15]. Lee et al. reported that iron overload increases intracellular calcium levels in HT-22 hippocampal neuron cells, thereby activating calcineurin, which promotes the dephosphorylation of Drp1 at Ser637, and thus enhances its recruitment to mitochondria and drives excessive mitochondrial fission [45]. Collectively, these findings underscore iron overload as a critical upstream driver of mitochondrial dynamics imbalance, initiating pathological processes that culminate in mitochondrial dysfunction.

Some studies indicate that iron overload may generate ROS, activating redox-sensitive calcium channels (such as ryanodine receptor, RyR) in the endoplasmic reticulum (ER), thereby inducing the release of Ca²⁺ from the ER and disrupting the mitochondrial network [57]. Recent studies have found that ROS can also promote the Small Ubiquitin-like Modifier-1 (SUMO-1) modification and de-Small Ubiquitin-like Modifier-2/3 (de-SUMO-2/3) modification of Drp1, damaging the mitochondrial structure in neuronal cells and impairing mitochondrial OXPHOS function [58]. Moreover, iron overload-mediated hyperphosphorylation of tau protein can disrupt the integrity of microtubules and interfere with mitochondrial transport [59, 60].

Mitophagy, a key quality-control mechanism for clearing damaged mitochondria, is regulated by both the ubiquitin-dependent pathway (PINK1/Parkin) and receptor-dependent pathways (e.g., BNIP3, FUNDC1), along with coordination with the lysosomal degradation system. Impaired mitophagy leads to the accumulation of damaged mitochondria, accelerating cellular aging [61, 62]. Fang et al. recently showed that in AD patient samples, mitophagy-related proteins PINK1 and BNIP3L/NIX are reduced, and autophagy initiation proteins such as p-ULK1 (Ser555) and p-TBK1 (Ser172) are inactivated. In AD-induced pluripotent stem cell (iPSC)-derived neurons, levels of other mitophagy-related proteins such as FUNDC1 and Bcl2L13 are also decreased, further confirming impaired autophagy in AD [63].

**Fig. 1:**
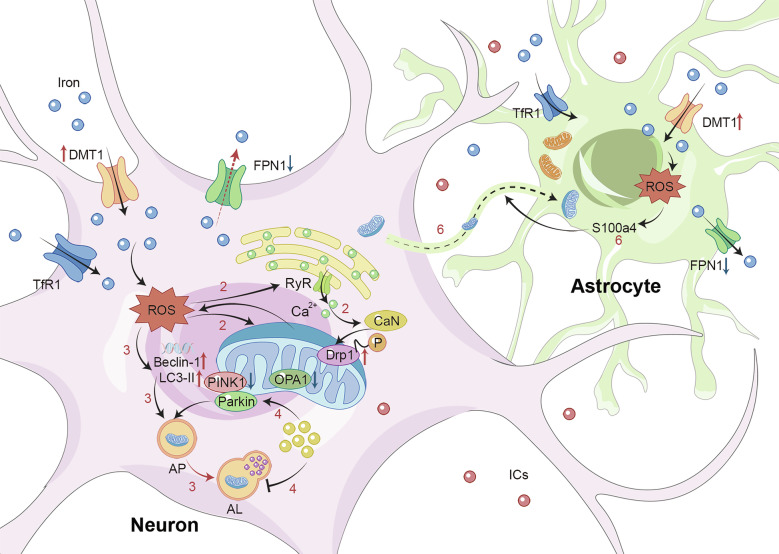
Iron overload disrupts mitochondrial dynamics and quality control. 1. Iron overload, oxidative stress, Aβ, and inflammation upregulate mitochondrial fission proteins (e.g., DRP1) while suppressing fusion proteins (e.g., OPA1) [15, 45, 143, 144]. 2. ROS generated by iron overload causes mitochondrial damage and activates calcium channels (e.g., RyR) in the ER, releasing Ca²⁺, which activates calcineurin, promoting mitochondrial fission [57]. 3. Iron overload enhances the expression of autophagy-related proteins (e.g., Beclin-1, LC3-II), promoting autophagosome formation but impairing fusion with lysosomes [67, 68]. 4. Aβ or iron overload can increase cholesterol levels in neurons, which induces mitochondrial damage signals (e.g., PINK1 accumulation) and promotes mitochondrial autophagosome formation. However, high cholesterol levels impair lysosomal clearance [10, 145, 146]. 5. Inflammation increases DMT1 expression in neurons and astrocytes, while reducing FPN expression, promoting iron uptake and enhancing iron-induced oxidative damage [97, 147]. 6.Oxidative stress upregulates S100a4 and promotes the formation of TNTs, resulting in the internalization and degradation of neuronal mitochondria by astrocytes, accelerating neuronal energy depletion [148]. The red dashed line indicates the inhibited pathway. Adapted from Chen et al. [141]. AL autolysosome, AP autophagosome, CaN calcineurin, ICs inflammatory cytokines, TNT tunneling nanotubes.

Several in vivo and in vitro studies have shown that iron overload, Aβ plaques, and tau phosphorylation are closely related to the decreased expression of these mitophagy-related proteins [64, 65]. Additionally, iron overload also leads to decreased expression of Bcl-2-interacting protein 1 (Beclin-1) and microtubule-associated protein 1A/1B-light chain 3-phosphatidylethanolamine conjugate (LC3-II), resulting in autophagy blockage [66]. In cases of acute iron exposure, ROS, as a potent inducer of autophagy, promotes the formation of autophagosomes. However, at the same time, iron overload interferes with the fusion of autophagosomes with lysosomes and inhibits lysosomal acidification and hydrolase activity, thereby blocking the autophagy process [67, 68] (Fig. 1).

## Iron overload and glucose metabolism disorders

The brain is a high-energy organ, consuming about 20–25% of the body’s glucose despite comprising only 2% of body weight. It primarily derives energy from glucose through glycolysis, the TCA cycle, and mitochondrial OXPHOS [69, 70]. Impaired glucose metabolism is an early hallmark of AD, with reduced glucose metabolism observed in patients with mild cognitive impairment (MCI) and AD before clinical symptoms appear [71]. Iron is an important regulator of glucose metabolism, and iron overload disrupts glucose metabolism in multiple ways, exacerbating neuronal energy crises [72].

### Iron overload induced brain glucose transport and metabolism disorders

Brain tissue heavily relies on glucose as its main energy source, with glucose first transported across the blood-brain barrier (BBB) by glucose transporter1 (GLUT1) and then into neurons by GLUT3 [73]. However, in human AD patients, the expression of both GLUT1 and GLUT3 is downregulated, indicating inhibited glucose uptake [74, 75]. Although the underlying mechanisms remain unclear, existing studies suggest that iron overload and ROS play crucial roles.

Excessive iron has been reported to cause oxidative stress and mitochondrial dysfunction, downregulating the expression of GLUT1 and key genes or proteins involved in glucose metabolism (e.g., G6Pase and Pck1). Oxidative stress can broadly interfere with enzymes involved in glycolysis, the TCA cycle, and ATP biosynthesis, ultimately leading to glucose metabolism disorders and reduced ATP production [70, 76]. Evidence indicates that oxidative stress suppresses the Phosphoinositide 3-kinase (PI3K)/Protein kinase B (Akt) insulin signaling pathway, leading to downregulation of GLUT1 and reduced glucose uptake [77]. Concomitantly, diminished AKT activity removes inhibition of FOXO3, thereby triggering an apoptotic cascade in neurons [78]. Moreover, oxidative stress perturbs key pathways such as HIF‑1α and AMPK, disrupting the expression and membrane trafficking of glucose transporters and further aggravating glucose metabolic dysregulation [79, 80].

As for interventions, the iron chelator M30 upregulates HIF‑1α and its glycolytic targets (e.g., aldolase A, enolase‑1, and GLUT1) in the frontal cortex of APP/PS1 mice, thereby improving neuronal glucose uptake and metabolic function [79]. Scavenging ROS likewise helps restore glucose metabolism. In N2a/APP695swe cells, the ROS scavenger N‑acetyl‑L‑cysteine (NAC) activates the PI3K/Akt pathway, significantly increasing GLUT1 expression, glucose uptake, and ATP levels while reducing Aβ levels [77]. In PC12 cells, ROS clearance promotes AMPK activation and alleviates defects in GLUT4 translocation, thereby ameliorating glucose metabolic dysregulation and inhibiting apoptosis [80].

### Iron overload induced brain insulin resistance

Converging evidence implicates dysregulation of the brain’s insulin signaling pathway as a key driver of AD, framing the disorder within the concept of “Type 3 Diabetes”—a state of central insulin resistance (IR), characterized by impaired insulin receptors (InsR)/insulin receptor substrate 1 (IRS-1)/PI3K‑Akt signaling and aberrant glycogen synthase kinase-3β (GSK-3β) activation [81].

Multiple studies support a close relationship between iron overload and impaired insulin signaling. do Nascimento et al. reported that neonatal iron overload induces systemic IR (elevated HOMA‑IR) alongside reduced hippocampal AKT phosphorylation, implicating iron in the disruption of central insulin signaling [82]. Wan et al. found that ferrous iron (Fe2+) increases tau hyperphosphorylation in primary neurons while reducing tyrosine phosphorylation of IRβ, IRS‑1, and the PI3K p85α subunit; similar changes and disrupted brain insulin signaling were also observed in iron‑overloaded mice [83]. These findings support a role for iron‑induced disruption of central insulin signaling in AD pathology. Furthermore, in obese individuals without diabetes, Blasco et al. found that peripheral IR (indexed by insulin AUC) was independently associated with brain iron overload (particularly in the caudate nucleus and hippocampus), and that brain iron overload was independently associated with poorer cognitive performance, suggesting that obesity and peripheral IR may impair cognition by exacerbating iron‑related pathology [84]. Supporting this notion, intervention with acarbose—a drug that ameliorates glucose homeostasis and reduces adiposity, significantly improved cognitive deficits in AD model mice fed a Western diet. Notably, these cognitive benefits were largely uncoupled from changes in tau or amyloid pathology, underscoring that enhancing systemic metabolism per se represents a powerful, complementary therapeutic avenue for AD [85].

Oxidative stress may play a key role in iron-mediated IR. In Ts65dn mice, increased oxidative stress was accompanied by reduced activities of InsR, IRS1, and AS160, occurring before APP-C99 accumulation. This suggests oxidative stress-mediated insulin signaling damage is an early event in AD development [86]. Syrovatka et al. found a positive correlation among ferritin, oxidized low-density lipoprotein, advanced oxidation protein products, and impaired insulin sensitivity in healthy males, confirming oxidative stress’s role in iron-induced IR [87]. In vitro, oxidative stress activates apoptosis signal-regulating kinase 1 (ASK1), triggering downstream effectors, including JNK1/2 and p38MAPK. These promote serine phosphorylation of IRS-1 and downregulate IRβ and AKT activity, impairing insulin signaling in AD patient-derived iPSC neurons [75]. Additionally, oxidative stress downregulates AKT activity, removing FOXO3 inhibition and activating it, further inducing neuronal apoptosis [78, 88].

**Fig. 2:**
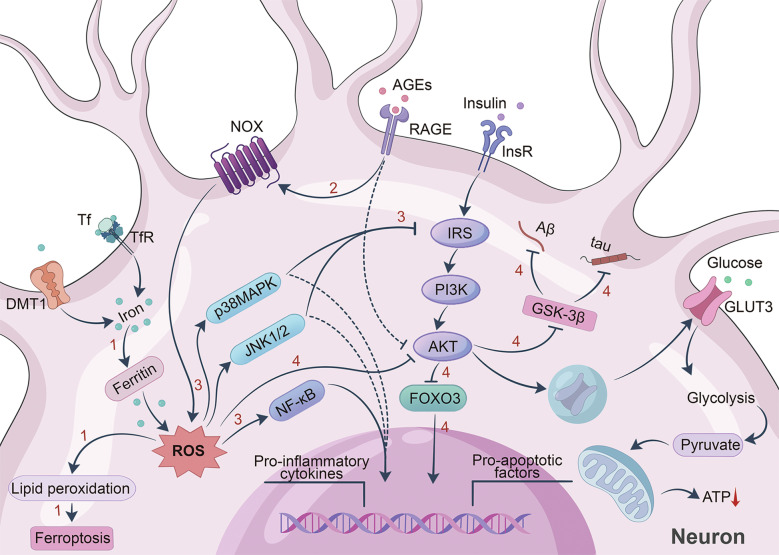
Iron overload impairs glucose metabolism in neurons. 1. Iron overload generates ROS through the Fenton reaction, triggering oxidative stress, lipid peroxidation, and even ferroptosis [2–4]. 2. Binding of AGEs to RAGE induces ROS generation, promoting oxidative stress [90, 91]. 3. Oxidative stress activates inflammatory pathways (e.g., NF-κB, JNK1/2, p38 MAPK), promoting inflammation and inhibiting insulin signaling [4, 75, 91]. 4. Increased oxidative stress and downregulation of AKT activity relieve the inhibition of FOXO3, activating apoptotic signaling [78]. Concurrently, GSK3β is activated, leading to increased Aβ generation and tau hyperphosphorylation [90, 91]. Adapted from Chen et al. [141].

Notably, advanced glycation end products (AGEs) accumulate abnormally in AD patients, closely related to oxidative stress and insulin signaling damage [89]. The binding of AGEs to the receptor for advanced glycation end products (RAGE) can induce NADPH oxidase-dependent ROS bursts and mitochondrial dysfunction, activating inflammatory pathways such as nuclear factor kappa-light-chain-enhancer of activated B cells (NF-κB) and c-Jun N-terminal kinase (JNK). Meanwhile, Akt activity is inhibited, accompanied by downstream activation of GSK3 and subsequent excessive phosphorylation of tau protein, forming a cascade of “oxidative stress-IR-Aβ/tau pathology,” thereby accelerating AD progression [90, 91] (Fig. 2).

## Iron overload mediates neuroinflammation and neuro-metabolic uncoupling

Iron overload disrupts brain energy metabolism and significantly contributes to neuroinflammation [3–5]. These events are not isolated but form a vicious cycle through bidirectional interaction—metabolic disturbances trigger neuroinflammation via multiple pathways, while neuroinflammation reprograms glial cell metabolism and inhibits mitochondrial OXPHOS, disrupting metabolic synergy between neurons and glial cells [6–8]. This ‘iron overload-metabolic imbalance-inflammation amplification’ cascade accelerates irreversible synaptic loss and Aβ/tau pathology.

### The positive feedback loop of iron overload and neuroinflammation

Iron, as a potential factor influencing neuroinflammation, has a complex mechanism closely linked to oxidative stress [3–5]. Kenkhuis et al. found that iron-loaded microglia did not exhibit typical pro-inflammatory or anti-inflammatory activation at the transcriptomic level. Instead, oxidative stress-related pathways, such as the NRF2 pathway, were significantly upregulated, accompanied by impaired mitochondrial metabolism and altered phagocytic function [92]. This suggests that iron treatment does not lead to acute pro-inflammatory activation of microglia.

In contrast, Nnah et al. found that iron overload promotes the inflammatory response of microglia, increasing the secretion of interleukin-1 beta (IL-1β), particularly in the presence of Aβ. This may be related to the increased generation of ROS, as well as the activation of NF-κB signaling and the inflammasome [93]. The use of ROS inhibitors effectively counteracted the enhancement of Aβ-induced IL-1β release by iron and prevented the activation of NF-κB and mitogen-activated protein kinase (MAPK) in microglia [93, 94], highlighting the critical role of oxidative stress in microglial inflammation. These divergent results suggest that iron overload may induce microglia into a non-classical, highly context-dependent activation state, the specific phenotype of which may be regulated by factors such as local oxidative stress levels and amyloid burden.

There is evidence that the inflammatory effects of iron overload interact with hypoxic signaling. Under hypoxic conditions, hypoxia-inducible factor 1-alpha (HIF-1α) exacerbates iron accumulation in microglia by upregulating iron transport proteins (such as TfR and DMT1), which in turn activates the p38 MAPK pathway, driving the production of pro-inflammatory factors (such as tumor necrosis factor-alpha (TNF-α) and IL-1β) [95]. Meanwhile, the endogenous anti-inflammatory pathway extracellular signal-regulated kinase (ERK)-cAMP response element-binding protein (CREB)-MAPK Phosphatase 1 (MKP1) fails to effectively inhibit p38 activity under iron overload conditions, leading to uncontrolled inflammation [95]. Targeting this mechanism, drugs aimed at HIF-1α or iron metabolism have shown anti-inflammatory potential [95, 96]. For example, HIF-1α inhibitors (KC7F2) and iron chelators (Deferoxamine, DFO) can reduce iron uptake by inhibiting TfR/DMT1 expression while enhancing the ERK-CREB-MKP1 pathway activity, thereby inhibiting p38 phosphorylation and reducing the release of pro-inflammatory factors [95].

**Fig. 3:**
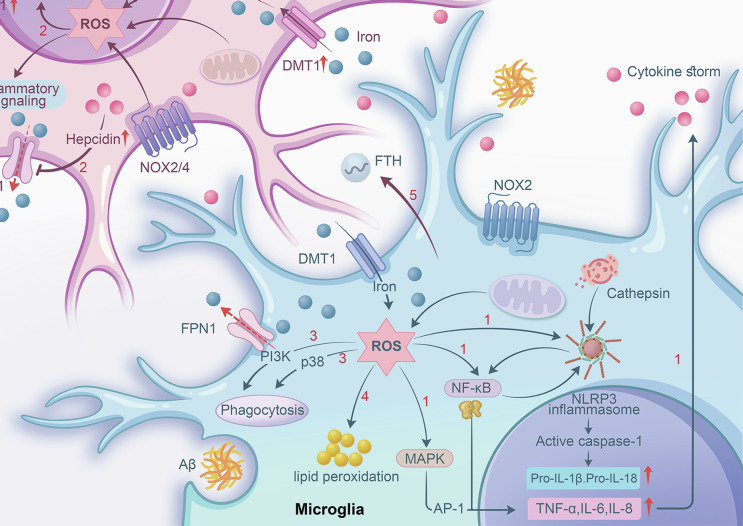
The iron-inflammation feedback loop in AD. 1. In the context of AD, increased ROS generation in neurons and microglia activates inflammatory signaling pathways, such as NF-κB and MAPK [4, 93, 94]. 2. Inflammatory factors such as IL-1β or TNF-α released by microglia can upregulate DMT1 expression while downregulating FPN1 expression by upregulating IRP1 and hepcidin. This exacerbates iron overload and ROS production within neuronal cells. This vicious cycle further accelerates oxidative damage to neurons [93, 98]. 3. Microglial phagocytosis depends on ROS-mediated activation of PI3-K and p38 MAPK [99], but activation of the NLRP3 inflammasome affects the clearance of Aβ by microglia [100]. 4. Excessive ROS production induces lipid peroxidation, amplifying inflammation and potentially triggering ferroptosis [3, 4, 101]. 5. Activated microglia release FTH via exosomes, stimulating membrane lipid peroxidation and neuronal ferroptosis [102]. The red dashed line indicates the inhibited pathway. Adapted from Chen et al. [141]. AP-1 activator protein-1, FTH ferritin heavy chain, GLS glutaminase, GLu glutamate, NLRP3 NOD-like receptor family pyrin domain-containing 3.

It is noteworthy that there exists a self-reinforcing positive feedback loop between iron overload and neuroinflammation. Studies have shown that inflammatory factors (such as TNF-α and IL-1β) can upregulate iron import proteins (DMT1) in microglia and neurons while inhibiting iron export proteins (Ferroportin 1, FPN1), leading to intracellular iron retention [97, 98]. Further research indicates that this regulatory process is closely related to oxidative stress mediated by ROS and nitric oxide (NO) induced by inflammatory factors: ROS/NO promotes DMT1 expression and inhibits FPN1 function by upregulating iron regulatory protein 1 (IRP1) and hepcidin, ultimately forming a vicious cycle of “iron overload-inflammation-iron retention” [98]. This cycle not only exacerbates oxidative damage to neurons but also severely impairs microglial function, promoting Aβ deposition and Tau pathology, accelerating the progression of AD [93, 97, 99–103] (Fig. 3).

The causal relationship between iron overload and neuroinflammation in AD remains a forefront research question. Clinicopathological evidence suggests that abnormal iron accumulation may not always be the initial driver of the disease but can, to a considerable extent, also be a downstream consequence of ongoing neuroinflammatory processes. For instance, a large-scale cohort study by Ayton et al. found that cortical iron burden was strongly associated with accelerated cognitive decline in individuals with AD pathology; however, this iron accumulation could also be interpreted as a result of neuroinflammation-activated microglia sequester iron, and upregulation of pro-inflammatory signaling pathways promotes intracellular iron retention, which is part of the host defense response [5]. This indicates that iron may act as an upstream instigator in certain disease stages or contexts, while in others it becomes a secondary response to inflammatory activity. Regardless, once iron overload is established, it itself constitutes a powerful amplifier of pathology. Subsequent iron toxicity exacerbates oxidative stress and cellular damage, thereby driving disease progression and functional decline [3, 5]. Therefore, the role of iron in AD is dynamic and complex, likely evolving with disease stages. This understanding provides a critical perspective for appreciating its multifaceted role and for designing stage-specific intervention strategies.

### Neuroinflammation-induced metabolic uncoupling

Neuroinflammation causes structural and functional damage to neural cells, particularly neurons, through immune cell activation and direct neuronal injury. It also significantly increases neural cell energy consumption and alters energy metabolism via metabolic reprogramming, disrupting the highly coordinated metabolic coupling between neurons and glial cells, further exacerbating metabolic disorders [104].

Under normal physiological conditions, neurons can utilize glucose, lactate, or pyruvate as metabolic substrates to generate ATP through aerobic glycolysis or oxidative phosphorylation (OXPHOS). Studies have shown that during neuronal activity, glucose is a more efficient energy substrate [105]. Under inflammatory conditions, when glucose availability decreases significantly and lactate production increases, neurons can adapt by switching to lactate as their primary fuel, demonstrating a certain degree of metabolic flexibility. However, this flexibility is limited: if the lactate/glucose ratio becomes too high, neuronal energy metabolism is impaired. Even with increased lactate supply, neurons still exhibit a significant energy deficit, ultimately compromising their function and viability [106].

Under both resting and activated states, neurons primarily rely on aerobic glycolysis for energy, with relatively low levels of OXPHOS. This helps avoid oxidative damage caused by excessive ROS production [107]. However, under inflammatory conditions, when neurons are exposed to factors such as interferon‑gamma and IL‑9, the gene expression of mitochondrial electron transport chain complexes, including ATP synthase, is suppressed, leading to a significant decline in mitochondrial respiratory function [108]. Due to their high energy demand and limited reserves, neurons are particularly vulnerable to mitochondrial dysfunction and bioenergetic deficits [109]. Reduced ATP levels impair the function of ion‑motive ATPases such as Na⁺/K⁺‑ATPase, resulting in membrane depolarization, overactivation of voltage‑gated Ca²⁺ channels, and ultimately cytotoxic Ca²⁺ overload [110].

Astrocytes play a crucial role in brain energy metabolism. Under inflammatory conditions, the metabolic pattern of astrocytes changes significantly, shifting towards a glycolysis-dominated pathway, which may be related to the activation of the NFκB and major histocompatibility complex class I pathways [111]. Astrocytes generate large amounts of lactate through glycolysis and secrete it extracellularly. Through the so-called “lactate shuttle” mechanism, lactate is transported to neurons via monocarboxylate transporters (MCT4), providing metabolic fuel to neurons, presenting an “inverse Warburg effect.” However, impaired lactate oxidation in neurons leads to inefficient utilization, resulting in intracellular lactate accumulation, insufficient ATP synthesis, and a consequent drop in intracellular pH (acidosis) that further inhibits mitochondrial function [7, 112]. Concurrently, acidosis activates acid-sensing ion channels, further exacerbating intracellular Ca^2+^ influx and triggering cell death pathways [113]. In vivo studies have shown that excess lactate from astrocytic glycolysis disrupts synaptic plasticity and accelerates Aβ aggregation, exacerbating cognitive impairment in APP/PS1 mice [114].

**Fig. 4:**
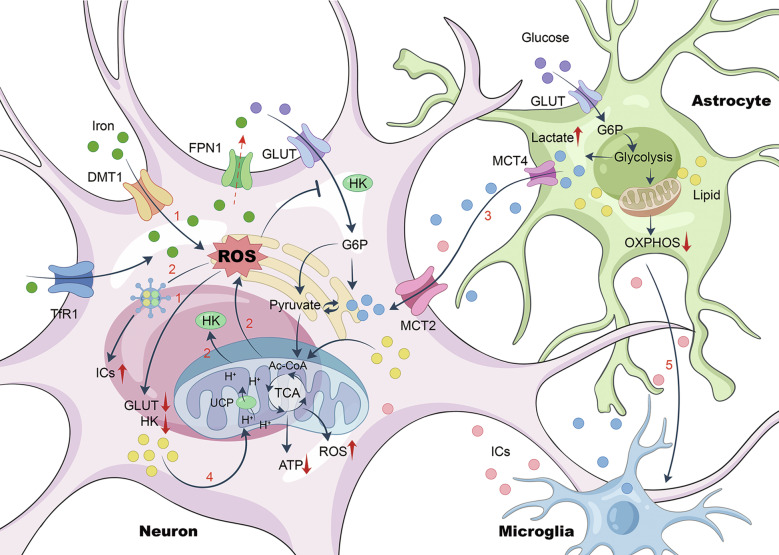
Iron overload disrupts neuronal energy metabolism. 1. Iron overload-induced oxidative stress activates inflammatory pathways and reduces GLUT and key glucose metabolism enzymes [70, 76]. 2. Inflammatory cytokines, such as IL-1β, inhibit HK expression and promote its detachment from mitochondria, increasing ROS and activating the NLRP3 inflammasome, worsening inflammation [149]. 3. Under iron overload and inflammation, glial cells switch to glycolysis, producing lactate for neuronal fuel. However, impaired lactate oxidation in neurons leads to lactate buildup and insufficient ATP synthesis [8, 111, 112, 116]. 4. Aβ or iron overload can lead to cholesterol accumulation in neurons and astrocytes [10, 145]. Astrocytes promote the hydrolysis of triacylglycerols in neurons, producing large amounts of FAs, which induce mitochondrial uncoupling and exacerbate the energy crisis [10, 150]. 5.Lipid-laden reactive astrocytes activate microglia through IL-3 signaling [151]. The red dashed line indicates the inhibited pathway. Adapted from Chen et al. [141]. Ac-CoA acetyl-CoA, G6P glucose-6-phosphate, HK hexokinase, UCP uncoupling protein.

Microglia, as the immune surveillants of the brain, have functions that are highly dependent on dynamic regulation of glucose metabolism: primarily relying on OXPHOS in a resting state and shifting to glycolysis upon activation to meet the energy demands for rapid synthesis of pro-inflammatory factors. However, the decrease in metabolic efficiency also leads to reduced chemotaxis and phagocytic functions of microglia [6, 115]. Under pro-inflammatory stimuli such as LPS or Aβ, the levels of DMT1 and ferritin in microglia are upregulated, increasing the uptake of non-transferrin-bound iron (NTBI). This change in iron metabolism, accompanied by increased glycolysis and reduced oxidative respiration, suggests that iron overload is related to metabolic shifts in microglia [116] (Fig. 4).

## Therapeutic strategies targeting the iron-energy metabolism axis

### Development and application of iron chelators

Given the crucial role of iron overload in AD pathogenesis, utilizing metal chelators to reduce excess iron in specific brain regions has garnered attention as a strategy to alleviate or even treat the disease. Common iron chelators include deferoxamine (DFO), deferiprone (DFP), and deferasirox (DFX). They function by binding excess iron to form stable complexes, thereby reducing intracellular free iron levels, mitigating oxidative damage and neuroinflammation, and have shown potential in preclinical models to reduce pathology and improve behavior [117–119]. Preclinical studies have shown that 8-Hydroxyquinoline derivatives such as clioquinol, HLA-20, and M-30 possess strong iron-chelating and free radical scavenging properties, demonstrating potential for treating neurodegenerative diseases [120]. Alpha-lipoic acid, an organosulfur compound with iron-chelating, antioxidant, and anti-inflammatory effects, has been shown to slow cognitive decline in AD patients when combined with omega-3 fatty acids [121].

However, the translation of iron chelation from compelling preclinical promise to consistent clinical benefit in AD has been challenging. This discrepancy may reflect multiple factors, including disease stage, heterogeneity in iron burden and form, and treatment precision [5, 19, 122]. Using brain tissue iron quantification by instrumental neutron activation analysis (INAA) combined with longitudinal cognitive assessment, Ayton et al. reported that, in individuals with AD pathology, elevated cortical iron burden was associated with faster cognitive decline, whereas in those with a low AD pathological load the trend was weaker or not significant [5]. Given that iron is essential for mitochondrial Fe–S cluster and heme biogenesis, myelination, and monoaminergic neurotransmitter synthesis, non‑selective iron chelation when iron burden is low, or iron availability is constrained, could theoretically precipitate or exacerbate functional iron deficiency and an energetic crisis [19, 23, 123]. This concern was starkly highlighted by a recent multicenter, double‑blind, placebo‑controlled randomized trial in early AD: oral DFP 15 mg/kg twice daily for 12 months reduced hippocampal Quantitative Susceptibility Mapping (QSM) yet, paradoxically, accelerated cognitive decline in the intention‑to‑treat analysis; exploratory regional analyses further suggested increased frontal volume loss [124]. Furthermore, stringent iron histochemistry together with transcriptomic analyses suggest that the AD brain contains a higher proportion of tightly bound/sequestered iron pools, and that anemia‑like and iron metabolism-related transcriptional changes emerge early, supporting a functional iron deficiency hypothesis; iron is hypothesized to be bound/sequestered by protein aggregates such as Aβ and tau, thereby reducing chelator accessibility and causing relative insufficiency of bioavailable iron [19]. Therefore, the therapeutic window for iron chelation may be narrow, with the risk–benefit strongly dependent on disease stage, the form in which iron is present, and the precision of dosing.

Pharmacokinetics and brain delivery represent another key translational bottleneck. DFO is highly hydrophilic, has low oral bioavailability, and poor BBB penetration; DFP has higher oral bioavailability, with some studies suggesting limited brain entry, but carries risks of neutropenia/agranulocytosis; DFX offers convenient oral dosing and a long half-life, but has limited BBB penetration and hepatorenal toxicity [120, 125]. The BBB disruption in AD itself is regionally heterogeneous and varies between individuals, further complicating predictions of brain exposure [126]. Targeting receptor-mediated transcytosis (RMT) is one strategy to improve brain delivery. However, drugs or nanoparticles targeting the transferrin receptor (TfR) face clinical translation hurdles due to peripheral tissue sinks, reticulocyte depletion, and lysosomal entrapment issues [126–129]. Strategies employing lower-affinity, monovalent binding can, to some extent, improve the brain delivery/safety trade-off [128, 129]. Notably, our team has successfully delivered iron-binding peptides into the brain by fusing them with ApoE or ApoB amino acid fragments, leveraging LRP/LDLR-mediated transport. This approach cleared excess iron and radicals, alleviating cognitive impairment in mice, suggesting that targeted delivery may enhance efficacy and safety [130–132].

To overcome these translational barriers and navigate the narrow therapeutic window, future clinical strategies must pivot towards precision medicine, focusing on: (1) Patient Stratification and Personalized Therapy: Utilizing iron-sensitive MRI techniques like QSM and CSF iron-related biomarkers for patient stratification to enable personalized, tailored, and adjustable chelation regimens that maximize net benefit and minimize the risk of excessive iron removal [123, 133]. (2) Minimizing Disruption of Essential Iron Pools: Prioritizing targeting of the labile iron pool (LIP) while avoiding mobilization of structural iron stores, thereby preventing functional iron deficiency and subsequent mitochondrial iron depletion and energy crisis [123, 134]. (3) Targeted Delivery and Site-Specific Activation: Ensuring brain delivery while controlling peripheral exposure, favoring targeted delivery systems and site/environment-activated prodrugs (e.g., ROS-responsive or target enzyme-activated) to confine activity to the pathological microenvironment, thus balancing brain access and safety [127, 129, 135, 136]. (4) Safety and Pharmacokinetic Monitoring: Incorporating routine monitoring of hematological, hepatic, renal, auditory, and visual function in clinical trials, combined with population pharmacokinetics and neuroimaging to assess brain exposure. Particular vigilance is required for DFP-associated agranulocytosis, DFX hepatorenal toxicity, DFO ototoxicity/ocular toxicity, and potential reticulocytopenia/anemia with RMT-based strategies [125, 127–129].

### Metabolic intervention strategies

Therapeutic strategies for improving energy metabolism defects in AD mainly focus on restoring mitochondrial function and improving glucose metabolism, thereby enhancing the energy supply to neurons. Common drugs such as Lacosamide and Methylene Blue improve mitochondrial function and promote energy production [137, 138]. Drugs like Acarbose and Metformin can improve glucose metabolism and enhance metabolic function [85, 139]. Antioxidant drugs such as vitamin E can reduce oxidative stress and protect mitochondria from oxidative damage, indirectly improving the energy supply to the brain [140].

However, current metabolic improvement therapies have some limitations: many drugs have limited effects on cognitive symptoms and significant side effects; existing treatment regimens often target a single mechanism and lack multi-target combined therapies; moreover, therapeutic effects vary greatly among individuals, and most drugs show better efficacy in the early stages of AD, with diminished effects in later stages [141]. Ketogenic diets, as a metabolic improvement strategy, provide alternative energy sources for the brain by increasing ketone body production [142]. Although they have shown some efficacy in animal models and early clinical trials, their long-term effects still require further research, especially considering individual metabolic characteristics and disease stages.

## Conclusions

AD is increasingly recognized as a multifactorial disorder, with iron dyshomeostasis and bioenergetic failure serving as its key, interconnected pathological drivers. A prevailing view in the field is that the role of iron in AD is not singular or static but exhibits high context-dependent complexity. It is both an essential physiological cofactor and a major source of oxidative stress. Available evidence suggests that iron overload may act as an upstream metabolic disruptor, triggering bioenergetic crisis and neuroinflammation; alternatively, it may arise as a downstream consequence, driven or amplified by ongoing neuroinflammation (e.g., iron sequestration by activated microglia) and proteinopathy (e.g., iron chelation by Aβ/tau), as indicated by clinicopathological studies. Regardless of its initial trigger, once established, iron overload becomes a powerful amplifier of pathology, fueling a vicious cycle that exacerbates oxidative stress, disrupts metabolic coupling, and promotes cell death, ultimately accelerating neuronal loss and clinical decline.

Therapeutic strategies targeting iron overload, such as iron chelators, show promise in preclinical models. However, their translation to the clinic is fraught with challenges, as evidenced by trials where reducing brain iron did not confer cognitive benefit and sometimes caused harm, starkly underscoring the non-linear, stage-dependent role of iron. This reality, alongside persistent hurdles like blood-brain barrier penetration and off-target effects, mandates a paradigm shift toward precision medicine. Future success will depend on biomarker-guided patient stratification (e.g., using imaging and fluid biomarkers) combined with stage-adapted interventions, whether through refined chelation protocols, multimodal approaches, or novel delivery systems designed to navigate the unique pathophysiology of the AD brain. Personalized therapy based on an individual’s iron status, metabolic profile, and disease stage will be crucial for improving outcomes.
